# DNA Origami and Their Application in Biosensors

**DOI:** 10.3390/bios16050247

**Published:** 2026-04-29

**Authors:** Iqra Nosheen Salim, Rebecca Reay, Christine Denby, Chris Halloran, Tien Anh Ngo, Jon Ashley

**Affiliations:** 1 School of Pharmacy and Biomolecular Sciences, Liverpool John Moores University, 3 Byrom way, Liverpool L3 3AF, UK; i.n.salim@2025.ljmu.ac.uk (I.N.S.); beckyr165@gmail.com (R.R.); 2 School of Nursing, Public and Allied Health, Liverpool John Moores University, 79 Tithebarn Street, Liverpool L2 2ER, UK; c.e.denby@ljmu.ac.uk; 3 Department of Molecular & Clinical Cancer Medicine, Institute of Systems, Molecular & Integrative Biology, University of Liverpool, Liverpool L7 8XT, UK; halloran@liverpool.ac.uk; 4 Institute for Regenerative Medicine, Hanoi 100000, Vietnam; tiennabio@gmail.com

**Keywords:** biosensors, DNA origami, diagnostics, design tools, DNA nanostructures

## Abstract

Biosensors have evolved significantly since their invention in the mid-twentieth century. From a simple electrochemical device to the current inclusion of AI, these sophisticated tools are capable of label-free, real-time multiplex detection. To make these sensing systems even more powerful, the incorporation of DNA origami has allowed this technology to become extremely precise, recognisable, and programmable to a range of molecules. This paper systematically summarises the incorporation of DNA origami with biosensors such as fluorescence, surface-enhanced Raman spectroscopy (SERS), surface plasmon resonance (SPR), and electrochemical sensors as well as approaches that are used to design DNA origami nanostructures. These tools allow a range of targets to be detected, ranging from small molecules to larger biological species. Collectively, these studies demonstrate that DNA origami-based biosensors provide high sensitivity; precise spatial control; and rapid, modular detection capabilities. Furthermore, their versatility enables applications across a diverse range of sectors. However, key challenges including limited reproducibility, structural instability, photobleaching, and non-specific binding continue to hinder their widespread adoption. This review proposes future directions aimed at overcoming key limitations, including enhancing biocompatibility and structural stability, to support the development of more advanced and clinical point-of-care-applicable biosensors.

## 1. Introduction

Biosensors are analytical devices that are capable of measuring biological or chemical reactions by generating proportional signals to the concentration of a chosen analyte in a reaction. The concept was first proposed in 1906 by Michael Cremer, who demonstrated that an electric potential was proportional to acid concentrations in a fluid across opposing glass membranes [1]. From this, Leland Charles Clark, Jr was able to invent the first true biosensor, an electrochemical biosensor, in 1956 regarding oxygen detection (the oxygen electrode is named after him—Clark electrode), which led to him being declared the ‘father of biosensors’ [2]. Table 1 shows how these discoveries have allowed a range of biosensors and nanosensors to be developed over the last 70 years.

One new advancement in biosensing that has started to gain traction is the incorporation of DNA origami (DNA nanostructures). DNA origami is a technique that was first introduced by Paul Rothemund in 2006. He demonstrated that custom 2D or 3D nanoscale structures can be constructed using only DNA. Long single-stranded DNA (ssDNA) molecules function as a foundational scaffold, as shorter ssDNA act as staples which bind to specific areas of the scaffold to allow folding to occur to create a desired shape through annealing [18]. His concept stemmed from the work of Nadrian Seeman, the father of nanotechnology, who first proposed the idea of using DNA as a self-assembling building material by obeying the Watson–Crick base pairing rule [19,20].

DNA origami has emerged as an exciting tool of interest in the fields of biomedical engineering, biotechnology and nanotechnology due to how it takes advantage of complementary base pairing, and a range of complex, stable structures (hearts, smiling faces and stars) can be engineered into precise shapes with defined sizes [21]. Secondly, it allows the incorporation of the bacteriophage M13 to function as the scaffold. This genome is a long single-stranded circular DNA, which is commercially available and also consistent in size (7249 nucleotides), making it well-identifiable [22]. From this, it can be used in computer-aided design tools such as caDNAno to automatically apply complementary staple sequences on a routing pathway where deemed appropriate [23]. Finally, the issue of stoichiometric ratios between the scaffolding and the staple strands is non-existent because it is encouraged to involve an excessive amount of smaller staple sequences to help reduce the possibility of mismatch and allows the self-assembly of the DNA to be more efficient by introducing more templates [24].

On the contrary, DNA origami has some challenges to overcome. To begin with, it is difficult to prevent DNA origami from denaturing under physiological conditions due to fluids naturally occurring within the body containing low concentrations of cations that are essential to keeping DNA origami stable, such as Mg^2+^ and Na^+^ [25]. Furthermore, these biological fluids also contain nucleases that can break down nucleic acids even in DNA origami which limits their in vivo half lives [26]. Another challenge that has been recorded stems from immunogenicity. As mentioned above, the scaffold M13 is most commonly used, yet it being derived from a bacteriophage genome can lead to an immune response, reducing the effectiveness of the DNA origami as well as creating adverse reactions [27]. This review aims to provide a focused overview of DNA origami-based biosensors, examining their design strategies, sensing mechanisms, and overall performance when compared to conventional biosensing approaches. Despite significant progress in this field, there remains a lack of review papers that combine the different types of design principles of DNA origami nanostructures with their practical applications and associated challenges in biosensor development. This review therefore addresses this gap by critically evaluating both the advantages and current limitations of DNA origami in biosensing, while also considering strategies to improve their stability, sensitivity, and real-world applications.

The paper is organised as follows: Section 2 explores the different types of DNA origami biosensors that are currently developed, as well as their advantages and disadvantages, especially when compared to their conventional counterparts. Section 3 discusses the tools and design approaches that are utilised to construct DNA nanostructures and highlights their strengths and weaknesses. Section 4 presents conclusions on the current state of this field and future perspectives on the key areas that need development to make DNA origami biosensors more reliable and practical enough to replace conventional biosensors.

## 2. Classes of DNA Origami Biosensors

### 2.1. Fluorescent Biosensors

The most common type of DNA origami-based biosensors that have been developed are fluorescent biosensors and these are summarised in Table 2. These tools have been praised due to their nature of being highly sensitive, their ease in reading results that are provided rapidly, and their use in both real-time analysis and endpoint measurements [28,29,30,31].

**Figure 1:**
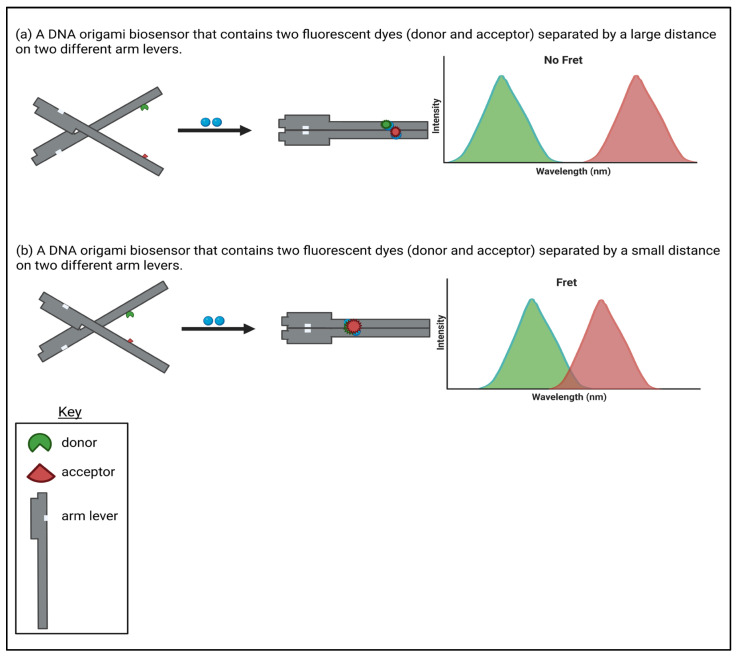
Fluorescent DNA origami biosensor. (**a**) The biosensor is triggered by allowing the two levers to close on each other; however, not enough energy is able to be transferred between the donor and acceptor leading to no FRET occurring; (**b**) the biosensor is triggered by allowing the two levers to close on each other, and this time enough energy is able to be transferred between the donor and acceptor leading to FRET reaction occurring. Created with BioRender.com.

As DNA origami can be designed, functionalised and precisely engineered, it gives this technique the opportunity to tailor and arrange quenchers, probes, and fluorophores spatially on a scaffold to emit or quench a fluorescent signal and act as transducers in biosensor design (Figure 1) [30]. A range of targets can be detected and quantified, such as nucleic acids, proteins, small molecules, and metal ions [32].

One example of a fluorescent biosensor, which was constructed to detect nucleic acids, was conducted by Zadegan et al. [33]. They created DNA boxes that had an operable lid that was able to open and close when it came into contact with a range of molecular keys that were complementary to breast cancer microRNA 223 and microRNA 30c biomarkers. With the help of Förster resonance energy transfer (FRET) spectroscopy, the group included the FRET pairs Cy3 and Cy5 and placed the dyes about 3 nm away from each other on two different oligos on the DNA origami scaffold. By including this FRET mechanism, when the appropriate RNA keys came into contact with the boxes, hybridising to the DNA fastener/probe sequence, the opening motion caused the FRET efficiency to decrease whereas when the closed motion occurred, the FRET efficiency was restored. Furthermore, the group studied how much of the DNA boxes they were able to recover. They tested this under three conditions: DNA boxes biotinylated on the outside, DNA boxes biotinylated on the inside and DNA boxes without biotinylation at all. They found that 65% of the DNA boxes could be recovered when it was biotinylated on the outside compared to the inside, where they were only able to recover 17% of the boxes. Having the DNA boxes with no biotinylation at all meant that only a minute amount of box was able to be recovered, emphasising how biotinylating the DNA boxes can allow for their recovery, purification and reuse. This also demonstrated an effective strategy to immobilise DNA origami structures on biosensor interfaces. Involving DNA origami in this study was advantageous because the programmable lid directly enhances the sensing performance. By controlling the opening and closing of the box, target microRNAs interact specifically with the probe sequences, producing a measurable FRET signal only upon complementary binding. This design reduces non-specific binding, as well as increasing the sensitivity and reliability of detection.

In the case of protein analytes, a fluorescent DNA origami-based nanosensor was demonstrated by Taghdisi et al. [34]. His group created a DNA prism structure incorporating a fluorescent aptasensor that was successful in detecting prostate-specific antigens (PSAs) through a method called direct fluorescence emission. The fluorochrome dye involved (PicoGreen) was useful in this case as it is known to produce high fluorescence efficiency of over 1000-fold when successfully interacting with double-stranded DNA (dsDNA) and, furthermore, has no fluorescent effect when free roaming. The results of this study demonstrated a strong fluorescent signal when a PSA was present in the aptasensor, compared to weaker fluorescence output in its absence. This suggests that the DNA nanostructure was successfully formed in the presence of PSAs that occurred due to PSAs triggering the release of a complementary DNA strand, which created the foundation of the prism structure. This led to the completion of the remaining parts of the DNA origami shape. In addition to the fluorescent dye, the dsDNA structure produced a strong fluorescence signal to be observed [35]. As well as that, the DNA prism biosensor produced a very linear concentration range between 200 pg/mL and 300 ng/mL, and the limit of detection (LOD) established for the PSA was 30 pg/mL. This further explains how fluorescent components can be used to confirm that detection is accurately occurring inside with a finely tuned biosensor.

An example of fluorescent biosensors being used to detect small molecules can be seen through the study conducted by Walter et al. [36]. They detected adenosine triphosphate (ATP) using a traffic light DNA origami system. They constructed the nanosensor by embedding a split aptamer (that was specific to ATP) into a DNA origami that was designed to have two arm levers. This split aptamer was able to act as recognition units in each arm lever. Each lever arm was functionalised with a pair of cyanine–styryl dyes, where there was a green donor dye and a red acceptor dye. An energy transfer was known to be present if the two ATP molecules were bound to the target and as a result, forced the two dyes to close the proximity between them making the levels parallel to each other. This conformational change led to a fluorescent shift from the green to the red region to be read out as it underwent a FRET mechanism. This DNA origami design presence optimises spatial control of the distance and orientation between the FRET dyes, with its programmable nanostructure directly enhancing sensing performance by producing a sensitive, distance-dependent optical readout when complementary binding has occurred. The change in the DNA origami was further confirmed through atomic force microscopy (AFM) and fluorescent colour ratios of acceptor intensity to donor intensity (IA/ID) in the presence and absence of the target molecule. Their data also produced a linear concentration that was sensitive to the DNA origami biosensor ranging between 0.10 mM and 1.00 mM ATP. Even though this range is limited in terms of sensitivity, the validity of the study is not fractured as the main reason of this research was to demonstrate the successful construction and function of a DNA origami biosensing device that was capable of ATP-induced closing and fluorescence readout. However, these platforms may not be suitable for practising biosensing applications in vivo as the detection range is insufficient for detecting low physiological ATP concentrations in vivo. Further optimisation would be required to improve this value and take it from its current proof-of-concept form to a device that can be used in a biosensing environment. This research emphasises the advantage of fluorescent biosensors and their capabilities in allowing DNA origami biosensors to be directly read.

The detection of metal ions is important to protect human and environmental health [37]. This is because, even though they are essential to maintain nutrient and biological levels, they are toxic and a huge source of pollution, especially heavy metals [38,39]. Researchers [40] recently demonstrated metal ion detection of mercury (II) (Hg^2+^) in animal meat products, which can directly spread to humans through consumption, by creating a DNA origami-based biosensor that was based on a rolling circle amplification hairpin structure. The group designed a nanoladder sensor of six complementary short chains where one exposed end was functionalised with a thrombin-binding aptamer (TBA) that could easily recognise Hg^2+^. The 5′ end was labelled with the fluorescent dye carboxyfluorescein (FAM), while the 3′ end was labelled with the quencher 4-([4-(dimethylamino)phenyl]azo) benzoic acid (DABCYL). This design allowed close proximity between FAM and DABCYL due to partial complementarity, which indicated a FRET mechanism as the donor and acceptor emission spectrum overlapped when observed through the fluorescent spectra and the ultraviolet absorption. In the presence of Hg^2+^, the fluorescent output signal decreased because the ion can induce TBA from forming a hairpin structure and altering the spatial relationship between FAM and DABCYL by detaching itself from DABCYL via fluorescence quenching. A linear range was produced regarding the Hg^2+^ and the fluorescent recovery rate between concentrations of 1 and 1000 nM, the LOD was determined to be 1.78 nM, and the limit of quantification (LOQ) was 2.0 nM. Recovery experiments of Hg^2+^ were also performed on tap water using the biosensor to determine it in a real-life scenario. Across low, medium, and high concentrations that were spiked, a recovery range between 97.5% and 109.7% was observed, demonstrating good accuracy of the biosensor. This study confirms the same advantages of using fluorescent functionality and transduction in DNA origami biosensors for small molecule detection.

A more advanced example of a fluorescent DNA origami biosensing platform was demonstrated in a recent study involving dynamically reconfigurable DNA origami crystals by Yan et al. [41]. In this system, octahedral DNA origami frames were assembled into 3D lattices and functionalised with multiple DNA hairpin structures that acted as “switches”. These hairpins were designed to respond to specific ssDNA inputs through toehold-mediated strand displacement, allowing them to go from a closed state to an open state under specific conditions when a Key-DNA strand was detected. As a result, the distances between adjacent DNA origami units could be altered, producing controlled structural phases within the crystal lattice. To observe these changes in the crystals, the researchers incorporated various fluorophore-labelled DNA strands (such as Cy3 and FAM), which generated visible colour changes under fluorescence microscopy depending on if the hairpins were activated or deactivated. The results showed that the system could switch between multiple distinct structural states in a highly controlled and reversible manner, with each state associating with a different fluorescence output signal. This dynamic behaviour represents a significant advancement compared to traditional on/off fluorescent biosensors with its DNA origami design being the core reason for these advantageous characteristics. This is because the high programmability and nanoscale precision allows for the orthogonal placement of multiple responsive elements within different spatial distances, allowing independent and simultaneous control over structural and optical responses. The precise spatial arrangement of hairpins ensures that multiple stimuli can be detected, improving sensitivity and specificity compared to more conventional designs. Moreover, it is versatile as multiple stimuli can be detected and processed within a single platform. This study highlights how DNA origami not only serves as a structural scaffold but also actively improves biosensor performance by enabling dynamic, programmable, and visually detectable responses.

Even though the majority of fluorescent DNA origami structures utilise FRET, another rapidly growing and popular method that can also be used is surface-enhanced fluorescence (SEF). This is because it is highly sensitive and selective, rapid, robust and can reduce photobleaching [42]. This technique relies on an interaction occurring between a fluorophore and a surface, usually plasmon-activated, that consists of metallic nanoparticles where it is hypothesised that when a fluorescent molecule is placed at a distance near these nanostructures, its emission and excitation are modified by the electromagnetic field, increasing the fluorescent signal [43]. This technique has been developed to detect a large range of targets, many of which have already been discussed above: metal ions [44], small organic molecules [45], proteins [46], DNA and RNA [47,48] and bacteria, viruses and toxins [49,50,51]. Compared to the traditional, fluorescent-based devices, SEF allows more amplified output signals to be generated at lower target and analyte concentrations, as well as improving the signal-to-noise ratio (S/N). This statement can be proved by a study conducted by the Tinnefeld research group [52], where they exploited the SEF concept to create a novel system to detect single-molecule, non-amplified nucleic acids that were specific to the antibiotic-resistant bacteria known as *Klebsiella pneumoniae*. Through trial and error, they agreed to arrange a nanopattern on a microfluidic chip, which integrated a fluorescent reader and a specific DNA origami, which was able to detect 600 molecules in a 100 µL sample volume, 200 ± 50 out of the 600 in a one-hour time frame. *The high sensitivity of the system, reflected by an LOD of 5 aM, can be associated with the precise spatial organisation of the DNA origami within the microfluidic environment, which increases the signal readout. Furthermore,* an improvement in the ratio between the signal and noise was confirmed as silicification was employed on the DNA origami structures to ensure they stayed stable in complex samples. These results are more favourable when compared to other conventional amplification-based systems.

Despite the clear advantages, DNA origami-based fluorescent biosensors have a couple of disadvantages, one of which is that they are prone to photobleaching. This is the photochemical change to a fluorophore or dye that is permanent due to a prolonged excitation and prevents a fluorescent signal from being produced as its structure is changed, so its ability to emit light is disabled [53]. Furthermore, analysis where there are multiple fluorophores overlaying each other can be a major disadvantage due to the phenomenon ‘colour bleed-through’. This is where one fluorophore colour spills over in a different detector that is designed to confirm a different fluorophore also present in the device. This overlapping in bleeding over can make it difficult to accurately identify each fluorophore, compromising the final data and its quality [54]. As well as that, this can limit the number of different fluorescent labels that can be included in complex experimental detection simultaneously, reducing high-throughput monitoring [55].

### 2.2. SERS Biosensors

Another major class of biosensor which utilises DNA origami is SERS (Table 3). This transduction method is able to enhance weak Raman signals by focusing on molecules that can be absorbed on metal nanostructures known as hotspots by amplifying energy from electromagnetics present on the metal surfaces [56]. This effect was first observed by a team of researchers at the University of Southampton [57] when they unexpectedly saw an increase in intensity on a Raman spectrum, which helped to distinguish between two pyridine absorptions with the aid of a silver electrode. As this can increase the sensitivity of a Raman signal by a factor of 10^10^–10^15^, detection can be observed up to the single-molecule level, while the fingerprint information can be categorised for qualitative analysis [58].

**Figure 2:**
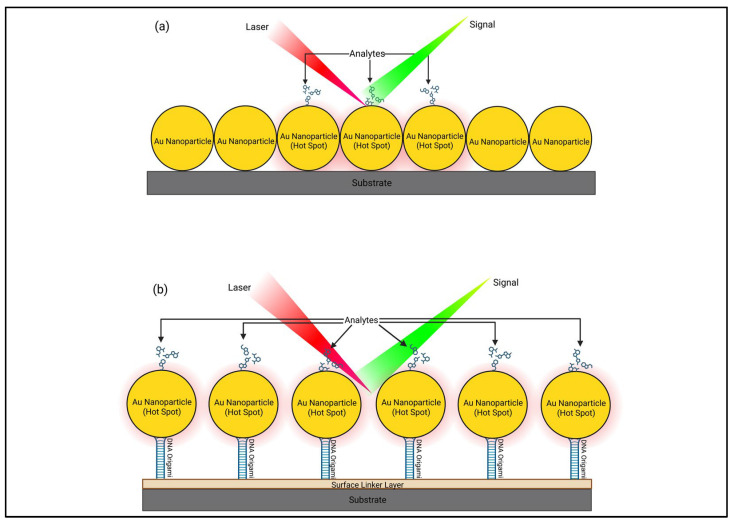
An illustration regarding a SERS DNA origami biosensor. (**a**) A traditional SERS mechanism which leads to a Raman signal being increased so that a local electromagnetic field is created, which allows this intensity to occur, leading to a strong signal being read which originally might have been very weak. (**b**) A SERS mechanism which involves DNA origami. This controlled, improved mechanism allows more hotspots to be generated with a laser and increases the localised electromagnetic field which allows a stronger signal to be generated. Created with BioRender.com.

Because DNA origami has a programmable design, the biosensors can be constructed beforehand with computer software so that a sensor can be created in a controlled manner that enables detection in a range of ways (Figure 2). For example, Prinz et al. [59] positioned gold nanoparticles that were coated with modified fluorescent dye 25 nm apart from each other, which led to the formation of Raman hotspots. This spatial arrangement improves the local electromagnetic field which therefore significantly increased the sensitivity of SERS. This is because closely spread nanoparticles enable plasmonic coupling where collective oscillations of surface electrons generate highly localised electromagnetic fields, hotspots, that amplify Raman signals. The intensity of these hotspots depends on nanometre gap distances. This demonstrates how the precise nanoscale control offered by DNA origami directly influences hotspot formation, which is a key factor of signal enhancement in SERS-based biodetection. As a result, the system achieved detection limits that exceed the commonly accepted S/N that equals 3, where the ratio is dimensionless as the calculations used are measured in the same intensity units. S/N was calculated as *(I_signal_ − I_baseline_)/σ_noise_*. The averaged spectrum obtained an S/N of 5.8 whereas the single-spot spectrum obtained a slightly lower S/N of 4.7, both of which are higher than the acceptable value. These findings indicate that DNA origami-based spatial control can produce more reliable and reproducible signal outputs compared to their conventional sensors. This study then later matured as more fluorescent gold particles were included, and the arrangement of the components was altered to detect the most desirable outcome. This further highlights the importance of design optimisation as constant alterations in these platforms can significantly influence the sensing performance. From this, DNA origami in biosensors has the ability to combine multiple signals that are close in proximity to create a cascade effect that results in a more significant signal from one single event that is more stable as well [60,61]. This cascade effect is advantageous when detecting low-abundance analytes due to the fact that amplified signals are generated without additional steps. Furthermore, this supports single-molecule sensitivity as DNA origami can position specific analytes directly onto these hotspots, ensuring that even the most minute of interactions produces a readable output signal.

As SERS is suitable in detecting low-abundance molecules, a range of studies have shown that SERS is able to differentiate between a range of molecules in complex mixtures of biofluids [62,63,64,65]. One example is shown by Sharma et al. [66], where they created gold nanorods and synthesised them onto a rectangular DNA origami to detect the biomolecule epidermal growth factor receptor (EGFR) in patients’ serum samples.

The use of DNA origami allowed precise spatial organisation of the gold nanorods, promoting the formation of consistent Raman hotspots. This demonstrates how nanoscale positioning enabled by DNA origami directly influences hotspot uniformity, which is important for generating reliable signals. Control such as this reduces variability in plasmonic coupling between nanorods, allowing consistent electromagnetic field enhancement across the sensor, which is essential for reproducible results in complex samples. This structural control provides reproducible results where Raman peaks were identified for the same molecules consistently staying in the same positions, also confirming uniformity in these complex biological samples. Their data was also ultra-sensitive as they determined an LOD of 0.2 nM, and their concentration range was between 20 nM and 20 pM. This enhanced sensitivity can be attributed to the controllable manner of the biosensor, which maximises the electromagnetic field enhancement at the hotspots.

The incorporation of SERS and DNA origami can also be used as a tool in the food safety industry. Researchers [67] wanted to target diethylstilbestrol (a non-steroidal synthetic environmental oestrogen) because it is known to be toxic if ingested; affects individuals’ reproductive and immune systems, pregnancies, and cardiovascular health; increases the risk of developing cancers; and is also an environmental pollutant. The team created a DNA origami out of triangles that included two gold nanoparticles, and when they used milk spiked with different concentrations of diethylstilbestrol, the device obtained a linear range from 10^−10^ to 10^−5^ M and LODs of different endocrine-disrupting chemicals ranged between 0.26 and 0.62 ng/mL and the same varied endocrine-disrupting chemicals produced LOQs of 0.8–1.9 ng/mL. These enhanced hotspots were determined to be highly sensitive and uniform [68], as well as sample recoveries in milk ranging between 90.6% and 108.8%. This demonstrates how the programmable nature of DNA origami influences the specific control over interparticle spacing, which is needed to generate consistent and reproducible SERS hotspots in complex environments such as this one. This particular spatial arrangement enhances local electromagnetic fields, thereby improving the sensitivity of SERS and contributing to the detection limits and linear ranges observed. In addition, by maintaining uniform interparticle spacing, DNA origami minimises signal variation between hotspots which improves the reliability of detection in heterogenous, real-world samples.

More recent and significant advances in DNA origami-based SERS biosensors can be seen in studies such as one demonstrated by Wu et al. [69], where they used a DNA origami SERS platform for imaging. They developed a SERS-based plasmonically coupled nanoprobe for targeted cancer cell imaging (SPECTRA) by assembling two gold nanorods into a dimer on a rectangular DNA origami template, forming a hotspot with a nanogap of 1.23 ± 0.48 nm. This precise spatial arrangement demonstrated how DNA origami can reduce the variability in hotspot formation, by providing reproducible nanoscale positioning to regenerate similar hotspots. This is important as conventional SERS substrates rely on random nanoparticle aggregation, leading to hotspot distributions that are heterogeneous and difficult to reproduce. The tightly controlled interparticle spacing resulted in strong plasmonic coupling, generating highly localised electromagnetic fields that amplified the Raman signal of a nitrile reporter by over three orders of magnitude compared to monomer constructs. This enhancement is seen because plasmonic coupling between closely spaced nanoparticles leads to electromagnetic field generation within the nanogap, with the intensity increasing as the distance between the gaps becomes more optimal. Beyond signal enhancement, the DNA scaffold also allowed systematic functionalisation with targeting aptamers, enabling simultaneous molecular recognition and further hotspot formation, which is particularly advantageous in differentiating cell types. Using this SPECTRA mechanism, an LOD of 20 pM was achieved for metastatic DU145 prostate cancer cells and a clear distinction was made between them and low-metastatic LNCaP cells, demonstrating both high sensitivity and molecular specificity. Overall, the design model emphasises that the structural programmability of DNA origami can control not only interparticle distance and orientation but also the spatial distribution of multiple hotspots, creating a cascade effect that enhances reproducibility, extreme sensitivity, signal stability, and the potential for multiplexing.

Another similar study that also wanted to image the same metastatic prostate cancer cells, DU145 and LNCaP, was conducted by Tanwar et al. [70] where they presented a novel Raman-active DNA origami (DO)-based hybrid nanodevice (ND) engineered for targeted drug delivery and imaging. The ND involved an enzyme-responsive peptide coating conjugated with the chemotherapy drug doxorubicin and cell-penetrating peptides, allowing selective uptake and activation in overexpressing DU145 cells while sparing low-expressing LNCaP cells. The precise spatial arrangement by the DNA origami scaffold allows optimal positioning of doxorubicin and Raman-active tags, enhancing optimised intracellular sensing performance. This spatial control is crucial, as positioning both the drug and reporter molecules near plasmonic hotspots increases the chances of generating a strong signal, and therapeutic action can also occur simultaneously at the same nanoscale location. This design introduces multifunctional integration, and ensures enzyme-responsive activation, collectively improving the specificity, sensitivity, and reliability of Raman imaging within target cells. Structural characterisation via microscopy techniques confirmed the protection of the DO structure after peptide coating, and stability assays demonstrated resistance to nuclease digestion, low salt conditions, and serum-mediated degradation. Raman spectroscopy was utilised to track intracellular localisation and monitor drug release, revealing a large distribution of the nucleus and cytoplasm of doxorubicin between DU145 and LNCaP cells, confirming selective activation. Furthermore, cytotoxicity studies emphasised this selectivity, as apoptosis only occurred in DU145 cells while having a minimal effect in LNCaP cells. Overall, the DO-based ND combines structural tunability, enzyme-responsive drug release, Raman imaging capability, and cell-specific cytotoxicity, representing a multifunctional platform with promising applications in precision cancer theranostics and future multimodal imaging strategies. The programmable DNA origami SERS device demonstrates precise spatial arrangement of the drug, enzyme-responsive peptides, and Raman-active tags, enhancing both selective activation and signal detection in a multifunctional nanodevice, allowing it to also commit to the combination of releasing targeted drugs, imaging capability, and cell-specific cytotoxicity within a singular platform.

Furthermore, Tanwar et al. [71] also investigated DNA origami-based SERS biosensors for their potential use in protein detection. In this study, silver-coated gold (Au@Ag) bimetallic nanostar (NS) dimers were assembled on rectangular DNA origami templates to generate specific plasmonic hotspots, which included programmable interparticle nanogaps of 5–10 nm enabling strong plasmonic coupling. By positioning a range of single reporter molecules such as FAM, Cy3, and Texas red (TR) within these nanogaps, the system achieved enhancement factors on the order of 10^9^–10^10^, supporting single-molecule sensitivity even under non-resonant conditions. This is important as single-molecule detection typically requires resonance conditions, but here the extreme electromagnetic enhancement generated within these nanogaps is able to compensate for the lack of molecular resonance. This highlights the role of DNA origami in hotspot engineering, where precise control over nanoparticle spacing and orientation produces reproducible and highly localised electromagnetic fields for signal amplification. This platform was further developed for protein detection, through the incorporation of aptamer-modified staples, allowing selective immobilisation of a single thrombin molecule directly within the hotspot. These results showed that SERS spectra displayed characteristic vibrational bands, including amide I and II and tryptophan signals, confirming label-free single-molecule detection. Furthermore, the plasmonic coupling between the NS tips created a broad and highly active enhancement region, creating strong signal amplifications even for relatively large biomolecules. Additionally, the system demonstrated broadband SERS capability, with maximal enhancement observed for Cy3 due to spectral overlap with the NS plasmon resonance, while specificity studies confirmed selective detection of thrombin over non-target proteins such as BSA and myoglobin. Overall, this work demonstrates how DNA origami’s structural programmability can achieve precise hotspot formation, controlled biomolecule positioning, and enhanced sensing performance, enabling reproducible, selective, and ultra-sensitive protein detection at the single-molecule level.

Finally, Kanehira et al. [72] demonstrated the use of DNA origami-based plasmonic antennas to determine its control over single-enzyme SERS detection. This was conducted by functionalising a single horseradish peroxidase (HRP) onto a DNA origami nanofork plasmonic antenna (DONA), where two gold nanoparticles were positioned to create a nanoscale plasmonic hotspot around the enzyme. Microscopy results confirmed the specific conjugation of HRP to the DNA bridge, with a height of 2.34 ± 0.37 nm, and the attachment of gold nanoparticles formed a DONAHRP, providing highly localised electromagnetic fields suitable for single-molecule SERS (SM-SERS) detection. Measurements analysed in liquid revealed characteristic HRP bands, including the ν_2_ (1550–1575 cm^−1^), ν_4_ (1362–1370 cm^−1^), and ν_10_ (1602–1631 cm^−1^) bands, corresponding to iron (Fe) oxidation and spin states, demonstrating reproducible single-molecule detection under physiological conditions. Furthermore, time series SM-SERS measurements tracked the HRP catalytic reaction where the addition of H_2_O_2_ converted Fe^3+^ to Fe^4+^, corresponding to compound I formation, while tetramethylbenzidine (TMB) addition generated signals for both TMB and its oxidised diimine form, reflecting the cyclic catalytic process. This capability highlights how the DNA origami scaffold not only positions the enzyme precisely within the hotspot but also enhances plasmonic coupling, amplifying Raman signals that are undetectable by bulk Raman. Moreover, the system captured peptide amide III bands, 1251 cm^−1^ for α-helix and 1235–1238 cm^−1^ for β-sheet, showcasing that DNA origami-enabled hotspots can probe the protein secondary structure at the single-molecule level. Overall, this DONA platform is an excellent example of the advantages of DNA origami due to its structural programmability by controlling the interparticle spacing, orientation, and functionalisation which directly influences hotspot efficiency, signal reproducibility, and sensitivity, which allows label-free, real-time monitoring of enzymatic activity.

Even though SERS is commonly used to detect biological and chemical molecules, there are several practical challenges for their widespread adoption and routine use. The main challenge is reproducibility and reliability. This is because the samples that are used are highly heterogeneous, and a lot of systemic error can result from sample preparation, which can all lead to variable signal outputs for the same target [58]. Expanding on that, obtaining a uniform signal is also difficult as there is usually a range of different hotspots that are generated, making this technique less dependable [73].

### 2.3. Surface Plasmon Resonance (SPR) Biosensors

**Figure 3:**
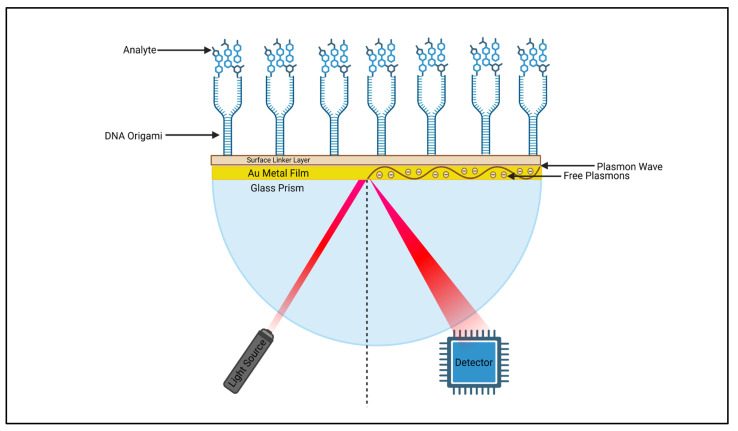
A schematic representation of an SPR DNA origami biosensor setup. A gold film has a light source directed at it through a glass prism. This generates the surface plasmons in the form of oscillating plasmon waves due to the free plasmons in the metal. The presence of DNA origami with the analytes that are immobilised onto the gold surface at precise and controlled positions shifts the angle of the reflected light, leading to the most optimal SPR signal to be monitored with a detector. Created with BioRender.com.

Another type of transducer which has been integrated with DNA origami is surface plasmon resonance (SPR) (Table 3). This is an optical, real-time system discovered by Liedberg [7] in 1983 when a surface constructed with a thin layer of gold (any thin layer of metal can be used) on a glass prism was exposed to polarised light. When the light hit the boundary between the glass and metal at a certain angle, the light was reflected at an angle known as the resonance angle. This angle allowed the light to interact with a range of free electrons that were present on the metal surface, leading surface plasmon oscillations to occur that can change the refractive index and have an effect on the original resonance angle, allowing a measurement to be detected and analysed in real time. This highly sensitive, versatile technique that is able to use a small amount of an unlabelled target for live monitoring [74] makes it popular for use in a range of fields such as drug discovery [75], the biomedical sector [76] and environmental studies [77] and why DNA origami is starting to be combined in the process to make devices even more sensitive and precise [78] (Figure 3).

The first reported combination of SPR and DNA origami was in 2019 by Shaw et al. [79], who decided to investigate how the spatial arrangement of antigens and antibodies has an effect on varied immune responses. They created two types of DNA origami, an 18-helix rod and a 44-helix brick, to have control over the distance between isotypes and antigens, ranging from 3 nm to 44 nm. Results show that all the antibodies that were tested bind bivalently when the antigens are spaced approximately 3 nm and 17 nm in distance. The most optimal distance they discovered was around 16 nm as this is where they noted the strongest binding affinity. Furthermore, the binding affinity was recorded to drop if the distance was too small (approximately 3 nm) or too long (approximately 17 nm). By involving SPR, precise *spatial arrangements were more controllable which led to appropriate interactions being captured* to help conclude immunochemical responses between a range of complementary pairings.

This integration has also been assessed for the detection of blood glucose levels. A group of researchers [80] working in the United States and Finland created a DNA origami cage to monitor blood glucose levels to help with the diagnosis of diabetes. One nanocage was designed to have a strand replaced with an aptamer that was responsive to either haemoglobin (Hb) or glycated haemoglobin (gHb). Three nanocages were created altogether: a cage that did not have any aptamers involved, a Hb-affinity aptamer (HA) DNA nanocage and a gHb-affinity aptamer (GHA) DNA nanocage. All three conditions were measured on an SPR sensor. It was found that binding and selectivity were more significant with the nanocages that were embedded with the aptamer compared to the DNA cage that was immobilised directly with the aptamer. This improvement can be linked back to the controlled spatial arrangements of the aptamers within the nanocage, which promote more effective target interactions. As a result, the HA-DNA cage and GHA-DNA cage showed 9 and 37 times higher binding towards Hb and gHb respectively. These results shows that it can be useful as a diagnostic tool to determine blood glucose levels in patients who might be suspected of having diabetes.

Daems et al. [81] also used SPR and DNA origami to arrange bioreceptors precisely around a nanostructure. A range of DNA origami structures was designed with varying configurations (8 nm to 113 nm) to position aptamers that were specific to thrombin. They utilised a fibre optic surface plasmon resonance biosensor (FO-SPR) and demonstrated the system in a range of conditions. It was determined that all the DNA origami structures had a high structural integrity as they maintained their shapes across pH 7–9 and with temperatures up to 60 °C. The reproducibility of the sensor was shown to be excellent as was achieved for FO-SPR, between 0.17 and 0.22 nm, confirming its ability to be repeated accurately. Near-perfect linearity was recorded across all the calibration curves (R^2^: 0.95–0.99), when tested on the thrombin concentration range. The LOD across the DNA nanostructures designs was determined to be between 6.1 nM and 11.2 nM. The high reproducibility, sensitivity and linearity can be directly linked to the spatial orientation aptamers on the DNA origami structures. The orientation was able to be controlled as two different sets of ssDNA, set 1 and set 2, were positioned on specific regions of the DNA origami. They looked at three specific distances, 8.5 nm (tetrahedron), 27 nm (lateral surface), and 113 nm (distal end), to determine how they affected the performance of the FO-SPR. The lateral surface DNA nanostructure produced the highest intensity signal at 7.74 nm, a very high correlation of 0.95, the strongest S/N of 18.9 and good reproducibility at 0.19 nm. However, the LOD calculated was the highest at 11.2 nm. The distal end on the other hand showed a reduction in the signal intensity down to 4.40 nm (which was the weakest) but produced the lowest LOD overall at 6.1 nm and a higher correlation of 0.98 nm in comparison to the lateral surface. The S/N produced was good as it came to 13.4. As well as that, it had the highest reproducibility of 0.17 nm. This improved performance is likely due to increased target accessibility at larger distances, demonstrating how spatial positioning of aptamers directly influences sensing efficiency. The final nanostructure, tetrahedron, produced the overall highest correlation of 0.99 nm, as well as good LOD concentrations and signal intensity as they were shown to be 10.7 nm and 4.68 nm. Unfortunately, this shape produced the lowest S/N of 9.8 and the highest reproducibility value of 0.22 nm. The researchers were able to conclude that precise control of aptamer spacing and orientation using DNA origami directly influences sensing performance, allowing their method to be reproducible, sensitive, and easily accessible, making it ideal for other investigators in similar fields to apply this to a wide range of diagnostic applications.

However, SPR is sometimes not able to differentiate between specific and non-specific binding in complex samples, requiring effective blocking agents, meaning a range of control samples may be required to produce more accurate data. It is also known to increase the noise-to-ratio background, which can produce false positive data by mistake [82]. Furthermore, the immobilisation of a molecule can lead to its biological properties altering due to conformational changes and denaturation occurring. This can also lead to inaccurate data being produced which can lead to misleading results [83]. These issues need to be resolved to ensure that SPR can be applied in different routine areas more confidently.

### 2.4. Electrochemical and Nanopore Biosensors

One alternative platform to previously mentioned devices is electrochemical biosensors, which are among the most widely used type of biosensor developed to this day as they are simple to operate, cheap, easily portable and have a short detection time [84]. These sensors work by immobilising a biorecognition element on the surface of an electrode. When the target analyte is recognised (e.g., binds or is catalytically converted) at the electrode interface, it causes a measurable change in an electrochemical signal such as current, potential, or impedance. The magnitude of this change is related to analyte concentration and is quantified using a calibration curve, allowing results to be visualised and interpreted [85]. These eye-catching features allow DNA origami to work well with electrochemical biosensors to further enhance their amplification, sensitivity, and versatility as shown in Table 4 [86].

A good example to represent this idea can be seen in the 2024 paper conducted by Jeon et al. [87]. They investigated how to make a versatile DNA origami electrochemical biosensor to detect a range of DNA and proteins. They did this by designing lily pads through DNA origami methods and enabled the ability to detect a range of molecules by replacing the binding domains where the analyte is placed for the target, by incorporating specific aptamers at precise positions on the DNA origami scaffold. In its open configuration, the lily pad holds the aptamers in a spatial arrangement that does not generate a strong signal.

Upon binding of a target molecule, in this case streptavidin and platelet-derived growth factor-BB, the DNA origami undergoes a conformational change, which “closes” the lily pad. This structural change alters the electrochemical properties at the sensor surface, producing a measurable signal that correlates with the presence of the target. The conformational changes were discovered to be more sensitive compared to other electrochemical sensors on the market. They determined an extremely low LOD of <1 pM and a log-linear range between 5 pM and 1 nM. The chip and sensor utilised also had the ability to be reused by regenerating them under mild conditions with only a ~5% loss in signal every time it was regenerated, showing how this DNA origami can be easily recoverable.

Another open and closed DNA origami electrochemical state biosensing device that has also been created was designed by Williamson et al. [88]. This two-part zipper structure contained 9 pH-responsive DNA locks that had the ability to open up at a pH of 8.0 and close when the pH was lower at 6.5. This DNA nanostructure was also able to be immobilised onto a gold surface by the addition of thiol groups. The different pH states of the zipper were able to be recorded appropriately through an electrochemical environment as during the closed state, the DNA zipper allowed fewer redox species to flow through the solution, which led to lower current peaks being formed and also a lower differential pulse voltammetry signal. When the zipper was open, the opposite occurred. The good performance of this sensor can be linked back to the DNA origami design because the precise spatial arrangement of the 9 DNA locks ensures that the zipper closes effectively under low pH, producing a predictable change in the electrochemical signal. The DNA origami scaffold also controls the structure consistently due to the position of the zipper on the gold surface, enhancing reproducibility and signal reliability.

DNA origami-based electrochemical biosensors may also be used to help protect the environment. Han et al. [89] created a 3D triangle nanostructure to aid in electron production for the *Escherichia coli* microbial fuel cells to improve the treatment of wastewater. This DNA origami would serve as an electron mediator–methylene blue carrier to improve the readily available technique. The team was able to successfully amplify the electron transfer between the bacteria and the electrode by involving the 3D DNA origami with methylene blue. An unmodified electrode maximum voltage was recorded at 37.8 mV, with a modified electrode with only methylene blue, the maximum voltage reached 53.4 mV and an electrode that was modified with both the methylene blue and DNA origami, the maximum voltage was 64.0 mV. This shows that DNA origami can be used with electrochemical biosensors to generate a greater voltage signal, and can be used to help protect the ecosystem. This is evident because the DNA origami precisely positions methylene blue on its scaffold for efficient electron transfer, which results in an increase in the maximum voltage generated by the microbial fuel cell.

A similar device to electrochemical biosensors that is rapidly becoming more popular is nanopore biosensors. They work by monitoring changes that occur to electric currents when a molecule is passed through a tiny hole (known as a nanopore) in specific membranes [90]. When a molecule is introduced to a nanopore, it partially blocks any current movement which leads to a disruption. This disruption is then measured and due to every molecule being unique, a specific squiggle signal (the characteristic current blockage signature/trace) is created and, as a result, is analysed. The results show information regarding the duration and amplitude changes that occur in the nanopore, allowing for single-molecule sensing to be achieved [91]. For this, nanopore-based sensors can be applied to a range of fields such as biomolecule detection, physics and environmental monitoring [92,93,94]. Involving DNA origami with nanopore sensing allows the shape and size of the pores to be precisely controlled, which can result in the translocation of molecules being influenced [95]. DNA origami has been applied to nanopore sensors in two ways: by trapping a nanostructure at the mouth of the nanopore or by inserting the DNA origami into a lipid bilayer.

An example of a nanopore sensor that includes a trapped DNA origami at the mouth was conducted by Joty et al. [96]. They wanted to investigate if they were able to increase the sensitivity of a solid-state nanopore for protein analysis and regulation by creating an octahedral DNA nanostructure. The DNA origami shape was confined inside a nanopore by electrophoretic trapping to allow their chosen single-molecule protein model, holo human serum transferrin (holo-hSTf), to undergo translocation in the biosensing nanopore across a range of electrolytic environments. These conditions varied depending on if the nanopore was combined with the DNA origami or not, and both nanopores were tested in 0.5 and 1.0 M of lithium chloride (LiCl) and potassium chloride (KCl) electrolytes. Furthermore, the voltage utilised was at a transmembrane potential of 200 mV. It was confirmed that DNA origami does play an important role in enhancing sensitivity, regardless of the changes that are occurring in the electrolyte concentrations. For example, in both the 0.5 and 1.0 M KCl, the hybrid nanopore consistently provided higher mean current blockages. This can be seen by how, in the 1 M KCl, the DNA origami nanopore recorded a value of 642.39 pA, whereas the pore that did not include the nanostructure in the same condition recorded a value of 178.78 pA. Similar results were recorded in the other electrolyte variables. This can be explained by there being stronger interactions between the holo-hSTf and DNA where DNA origami was present because the scaffold positions the proteins inside the pore in an orientation which amplifies the current blockage producing a stronger, more detectable signal and improving sensitivity across different electrolyte conditions, indicating that the use of a DNA origami trapped in a single-molecule sensor is advantageous to allow sensitivity to be enhanced.

An example of a nanopore sensor that inserts a DNA origami into a lipid bilayer was conducted by Wen et al. [97]. They created a nanopore electro-osmotic trap (NEOtrap) that allowed individual proteins to be captured and investigated. This label-free, single-molecule sensor consisted of a sphere DNA origami nanostructure that was locked onto a lipid bilayer by combining cholesterol molecules to its surface. Specifically, one cholesterol molecule was attached to each corner of the icosahedral structure bringing the total number to 12 molecules. Cholesterol molecules were used to attach DNA origami to bilayers of lipids because they have a very strong interaction to the amphiphilic molecules that are present in the lipid bilayers, such as phospholipids [98]. This results in an anchor effect between the DNA origami sphere and the nanopore. To test the full potential of this NEOtrap, tests were conducted on this cholesterol origami sphere nanopore vs. an origami sphere nanopore that was not coupled with the cholesterol molecules. The findings presented showcased that the entrapment and sensitivity of detecting proteins was enhanced with the cholesterol origami sphere nanopore. This is because a 100-fold increase in trapping time was recorded, and sensitivity of the proteins was measured to be as small as 13.7 kDa and increased all the way to 340 kDa in size. Furthermore, the S/N (⟨Δ*I*⟩/σ with Δ*I* = *I*_open_ − *I*_dock_) also increased to a reported value of 32.5; however, without the cholesterol the value was measured at 21.5 (a ~40% lower current-noise standard deviation). This shows how DNA origami embedded in lipid bilayers can be utilised because the DNA origami scaffold, anchored via cholesterol in the lipid bilayer, positions the proteins inside the nanopore in an orientation which amplifies the current blockage producing a stronger, more detectable signal. This improves sensitivity, trapping efficiency, the S/N and the detection of molecules similar to proteins when compared to conventional devices.

A more advanced example of a nanopore DNA origami biosensing platform was demonstrated in a recent study involving another type of dynamically reconfigurable DNA origami device which was conducted by Long et al. [99]. In this study, researchers designed a reconfigurable DNA origami hinge that changed between a closed and an open conformation state upon recognising a specific microRNA biomarker, miRNA-141-3p, which is associated with prostate cancer. The hinge is built of two flexible DNA origami arms which included Alexa488- and Atto550-labelled oligos on the bottom of the arm, and a quencher attached to the top of the arm. This hinge was also connected by a programmable strand displacement latch that allows it to remain closed. This locked state can be disturbed through a toehold-mediated strand displacement reaction which relaxes the latch and allows both of the hinges to extend when the target miRNA is detected. During solid-state nanopore translocation, the conformational change produces ionic current signatures, which allow individual origami carriers to be detected by their current amplitude and dwell time as they cross the nanopore sensor. This leads to a clean electrical signal being proportionally produced, as well as a signal amplitude and duration corresponding to hinge conformational change. The nanopore biosensor’s high sensitivity and reliability are a result of the DNA origami hinge’s programmable geometry and predictable mechanics. This nanosensor is able to thrive due to its DNA origami design: by controlling the spatial arrangement and flexibility of the hinge precisely, the origami ensures that the conformational changes recorded provide reproducible ionic current changes, allowing accurate detection of the target miRNA.

Even with all of the attractive features these electric current-operable sensors have, there are still a range of limitations these detection tools need to overcome. They both undergo interference from complex samples leading to inaccurate results, the stability can decrease due to degradation and harsh conditions, and selectivity can be poor as some of these biosensors can struggle to detect a target analyte with other similar molecules [100]. Specifically with electrochemical sensors, degradation can also affect the reproducibility which can lead to a loss of signal over multiple uses of the same molecule [101]. In regard to nanopore sensors, this device can be limited as it is difficult to control the speed at which molecules undergo translocation to the pores due to the process of diffusion naturally occurring [102].

### 2.5. In Vivo Biosensors

**Figure 4:**
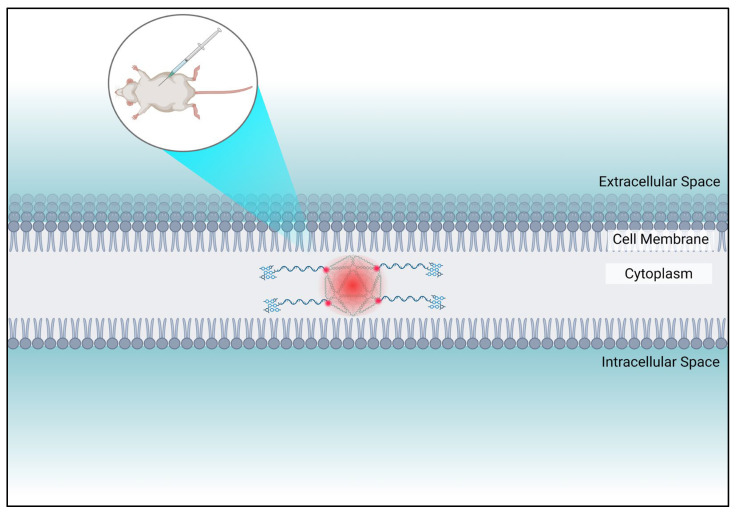
A schematic illustration of an in vivo DNA origami biosensor. A DNA origami nanostructure is administered by injection into a mouse model. Following delivery into the animal cell membrane, the DNA origami localises itself within the cytoplasm. The nanostructure is functionalised with ssDNA extensions protruding from the origami shape where the target molecules are attached to the termini of each individual ssDNA. Furthermore, fluorophore probes are also incorporated into the DNA design, allowing fluorescence-based signal generation upon molecule interaction, thereby allowing an intracellular target analyte to be detected and monitored within a real living organism’s cell. Created with BioRender.com.

Among the various alternative biosensing approaches, in vivo biosensors have gained significant importance due to their ability to continuously monitor analytes within a living organism in real-time, making them crucial for biomedical research, diagnostics, environmental and food safety monitoring [103,104]. Unlike the type of biosensors that have been mentioned previously, these devices provide constant data on how living systems respond to environmental changes not only at tissue and cell sites but also focusing on the full picture of a real, whole organism, allowing individuals and plant health to be surveilled to determine their condition [105]. In vivo biosensors are very flexible as they are able to be categorised by their bioreceptor (antibodies, enzymes and nucleic acids) or their transducer (optical and electrodes) [106,107]. DNA origami has been incorporated with in vivo sensors, enhancing the accuracy of individual measurements of target analytes [108] (Figure 4).

An example of an in vivo biosensor was demonstrated by Wu et al. [109]. They demonstrated that an origami-based bactericide can be created to aid in effective wound healing. Due to the organisational nature of DNA origami, DNAzymes (G4/hemin) were able to be placed in specific places on the nanostructure to controllably generate reactive oxygen species successfully. This spatial arrangement maximises bacterial membrane disruption and ensures effective delivery of the antibiotic, levofloxacin. This enhances the overall bactericide performance by allowing the levofloxacin to be delivered easily into the bacteria (also through the same DNA origami nanostructure) to allow sterilisation to occur so that the process of wound healing could be triggered. A range of conditions were tested on mouse models to determine how fast wound healing could occur over 12 days, for example, PBS only, DNA origami with only the G4/hemin, only the antibiotic levofloxacin in the form of a cream, and the origami-based bactericide which contained both the G4/hemin and the levofloxacin antibiotic. The results showed that the origami-based bactericide allowed wound healing to be more effective compared to any other group. From day 7, it recorded the highest wound closure and the scab wound had been completely removed naturally, whereas it took the whole 12 days for the same consequence to occur in the PBS-only group. Furthermore, at day 9 the area of the wound had rapidly decreased to 8.74% with the origami-based bactericide. This shows that this device is able to promote rapid wound healing and allows tissues to be regenerated more effectively in vivo. This nanoplatform could be easily integrated with a wound-monitoring system.

The research around in vivo biosensors that incorporate DNA origami is very limited. This is due to these types of investigations requiring large apparatus and heavy observations, and that it is highly controversial to do testing on animals, making it more costly [108,110]. Nevertheless, the majority of DNA origami in vivo biosensors are instead experimented on cell lines and human plasma [111,112] to help replicate human biology and physiology as they are more accurate compared to animal models.

### 2.6. Multiplex Biosensors

**Figure 5:**
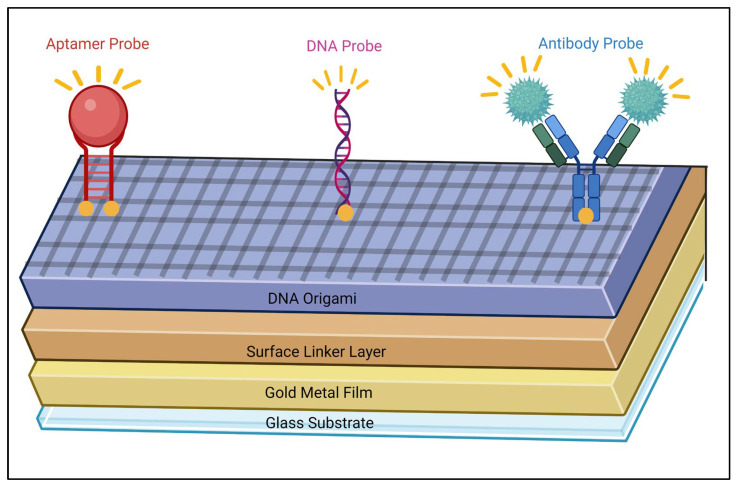
Schematic of a multiplex biosensor illustrating the immobilisation of multiple biorecognition elements on a layered platform. DNA origami is assembled on a gold film via a surface linker, enabling precise spatial arrangement of aptamer, DNA, and antibody probes for simultaneous, sensitive, and specific detection of diverse analytes. Created with BioRender.com.

Compared to previously discussed biosensing devices, multiplex biosensors are very important as they allow numerous analytes to be measured simultaneously in a given platform [113]. This allows a more in-depth analysis to be determined for diseases compared to a single-analyte detection biosensor, especially if the disease is highly complex. They are attractive devices that are predominantly used in the healthcare and medical sector due to the fact that they enhance reproducibility and reliability, they allow smaller sample volumes to be used and less materials are required overall, which reduces costs and saves on time on the whole, which is important for point-of-care tests [114]. Involving DNA origami allows these multiplex biosensors to be more sensitive when detecting their target analytes in a highly controlled and organised environment (Figure 5).

One way this idea has been explored was shown by Domljanovic et al., where they created a DNA origami book to help detect nucleic acid biomarkers associated with cancer such as miR-21 and let-7a [115]. To determine the effectiveness of making the biosensor multiplex, this DNA origami sensor either had fluorophores (at a distance of 5.8 nm) that were able to read and produce a fluorescent signal for either single-analyte detection or dual-analyte detection in 10 min. As a control, a non-DNA target was introduced to the single-analyte detection biosensor which resulted in a FRET efficiency to the left side of the book of ~8% and ~6% to the right side of the book. When a DNA target (ODN-153) was introduced, a significant change was observed as the FRET efficiency to only the left side of the book was ~26–30% at a concentration range between 100 pM and 1 μM, while a smaller change was seen at 10 pM at around ~10%. When another DNA target (ODN-342) was introduced, a significantly faster change was observed for the FRET efficiency to only the right side of the book when compared to the left side. For the same concentration range, the read output was around ~20% and at 10 pM it was reduced again to ~11%. For the ODN-153 and ODN-342 DNAs, their LODs were calculated at 1.6 pM and 1 pM. For the multiplex biosensor, both of the DNAs, ODN-153 and ODN-342, were measured in the concentration range 10 pM to 10 nM. The results gathered determined that the intensity of the fluorescence increased from ~12 to 20%. Furthermore, the fluorescence intensity remained at ~10% at the concentration of 10 pM, and a linear concentration range for the multiplex biosensor was achieved at between 1 and 10 pM. To determine if this biosensor book could detect specific nucleic acids linked to cancer, the same principle was adopted but with two highly expressed miRNAs instead, miR-21 and let-7a, at a concentration range of 10 nM and 10 pM. These two miRNAs were extracted from MCF-7 breast cancer cell extracts that were provided by patients diagnosed with breast cancer. The researchers found that at 10 nM the fluorescent intensity was increased to ~19% and at 10 pM the intensity was ~10%. This showed that the DNA origami book was able to detect real cancer-associated miRNAs and synthetic oligonucleotides when basing detection on fluorescent outputs in a 10 min time frame, as the scaffold increased the sensing performance by positioning fluorophores at specific nanoscale distances to maximise the FRET efficiency and produce measurable signals. Its modular design also enables multiplex detection by producing specific and sensitive results.

Another multiplex detection tool that has been created was done by Qu et al., where they demonstrated the multiple detection of heavy metal ions in water samples extracted from a contaminated river [116]. This team designed a 3D tubular tetrahedron DNA-nanostructured microarray that was able to recognise Hg^2+^, silver(I) (Ag^+^) and, lead(II) (Pb^2+^) within a 5 min time frame in the same device. For the heavy metal ion Hg^2+^, a linear concentration range was able to be seen at 10 to 200 nM and an LOD was calculated to 10 nM. For the heavy metal ion Ag^+^, a linear concentration range was able to be seen at 10 to 400 nM and an LOD was calculated to 10 nM. Finally for the heavy metal ion Pb^2+^, a linear concentration range was able to be seen at 20 to 2000 nM and an LOD was calculated to 20 nM. These results demonstrate how involving the DNA nanostructure allows the sensor to produce results that are rapid and sensitive when testing real-world samples. This enhanced performance is associated with the DNA origami scaffold, which was capable of precisely positioning multiple recognition sites for their targets in a rigid 3D arrangement, allowing rapid, sensitive, and simultaneous detection of multiple heavy metal ions in complex environmental samples.

Furthermore, a multiplex detection device created by Shahhosseini et al. [117] demonstrated molecular interactions in engineered DNA origami cells that are present in the 3D collagen scaffold. The researchers created a DNA origami cell sensing platform (CSP) that consisted of 40 dsDNA helices positioned into a multilayered structure with a central gap region and multiple ssDNA overhangs that serve as binding sites for specific target DNA sequences associated with interactions involving CH12-LX B cells and MutuDC 1949 dendritic cells, as well as the fluorescent dyes Cy3 and Cy5. By arranging these overhangs in precise spatial patterns, this platform allows simultaneous recognition of at least two different DNA sequences simultaneously. This mechanism works by using a fluorescence displacement strategy, where initially each binding site is hybridised with a modified quencher strand, keeping the fluorescence signal inactive until appropriate target binding occurs. Once target binding is detected, the quencher strand is displaced, which results in a measurable fluorescence signal. Due to the fact that multiple reporter strands are positioned in close proximity, the detection signal is amplified which allows multiple enhanced readout signals to be detected at once. Furthermore, the multiple layers increase structural rigidity by restricting the flexibility of individual strands and therefore this rigid and compact nanostructure allows this collagen scaffold to remain stable whilst having appropriate interactions. This CSP combines multiplex target detection and spatially controlled probe placements, making it an advantageous demonstration of a DNA origami scaffold that links a nanoscale design to a functional, real-time multiplex sensing performance.

A more recent multiplex DNA origami biosensor study by Sun et al. [118] presents a device that combines triangular DNA origami modules to help detect nucleic acids through toehold-mediated strand displacement. These triangles are incorporated with edge-specific hybridisation sites that are complementary to lung cancer biomarkers, specifically miRNA-155, miRNA-182 and miRNA-197. These hybridisation sites also allow the triangles to perform Boolean logic operations such as ‘YES’, ‘AND’, and ‘OR’ which in return creates a target-driven hierarchical self-assembly. This means that when a particular miRNA target is present, it binds to its complementary site on the DNA origami triangle, which triggers multiple triangles to assemble into a larger structure in a controlled manner. The programmability of the triangles confirms that only the correct combination of miRNAs leads to the correct assembly pattern, limiting the risk of false positives and cross-reactivity occurring in this multiplex detection. In return, a proportional structural change can be measured and visualised using microscopy techniques. This is a highly specific sensing device as each target miRNA is able to be detected and command a particular assembly pattern without being mistaken for a different miRNA and, furthermore, the modularity facilitates simultaneous detection of multiple biomarkers in a singular assay. By arranging the binding sites in specific positions at nanoscale sizes on the DNA origami scaffold, the sensor can accurately differentiate between multiple targets while maintaining high sensitivity. The precise nanoscale positioning provided by DNA origami allows the binding sites to be placed in specific placements on the scaffold, allowing accurate responses to be recorded for each target, or even targets. Overall, this study showcases how the programmability, spatial precision, and modularity of DNA origami are able to create a multiplex biosensor that is highly specific, sensitive, and capable of evaluating complex biomolecular information, such as structural and complementary changes.

Aiming to create multiplex biosensors does come with a range of limitations, the most obvious being cross-reactivity [119]. It can be difficult to analyse a range of targets within the same platform and ensure that the results being produced for each singular analyte are accurate—especially when creating a multiplex sensor for samples that are not highly specific; for example, polyclonal antibodies are not as specific compared to monoclonal antibodies [120]. Another major issue can be broken down to the overall sensitivity and specificity of these devices as multiplex biosensors tend to be less specific and sensitive compared to single detection biosensors [121]. This issue can be linked back to cross-reactivity but has also been seen through the interference of signals. The more signal outputs that are read simultaneously, the higher the chance they are likely to overlap with each other. This overlap can reduce the S/N. Both limitations can cause false positives and/or negatives, reducing the overall accuracy of the multiplex biosensors [122].

### 2.7. AFM-Based Biosensors

**Figure 6:**
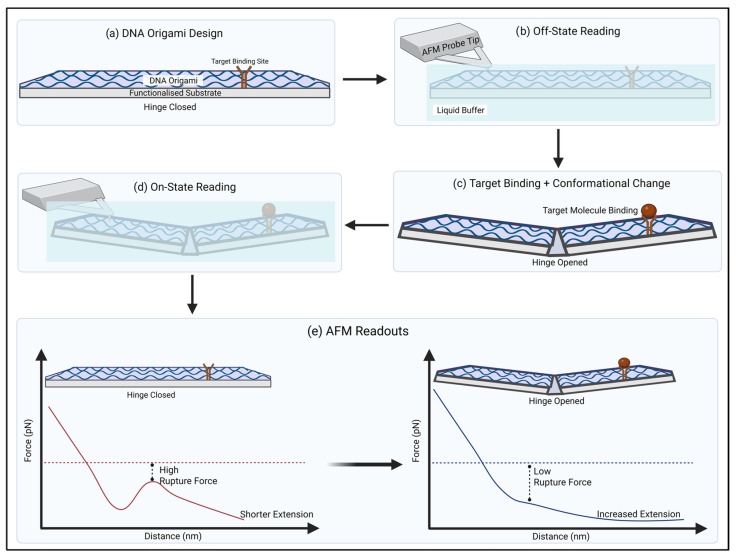
AFM-based DNA origami biosensor and force–distance readout. The closed hinge structure is imaged in liquid buffer in the unbound (off) state and re-imaged after target binding (on state), which induces hinge opening. Force–distance measurements demonstrate that the closed hinge exhibits higher rupture force and shorter extension distance, while the open hinge shows lower rupture force and increased extension distance due to reduced structural stability. Created with BioRender.com.

An alternative concept to DNA origami sensing involves coupling it to AFM readouts to enable label-free, single-molecule detection by directly imaging binding-induced structural changes. AFM measures nanoscale surface topography (and, depending on mode, mechanical interactions) by scanning a sharp probe across the sample. In origami-based assays, target binding can rigidify or reconfigure designed single-stranded features, producing a measurable change in AFM images that can be counted or quantified [123]. This label-free technique works by detecting interactions for biological molecules by measuring changes in the target’s height, force, or structural configuration by scanning their surface with a small, sharp probe [124] (Figure 6).

The most noticeable work done in this area was conducted by Ke et al. [125] in 2008 where they developed a DNA origami nucleic acid probe to successfully detect RNA at a nanoscale level. The team created a rectangular tile which contained three probe sets targeting transcripts expressed in a murine progenitor B cell line (Rag-1, c-myc, β-actin), and a non-target control was also included that was not expressed in the same manner. Each probe was copied twelve times in the DNA origami shape where they were spaced 5 nm in a line. Furthermore, each probe line was separated with a distance of 20 nm between them. These single-stranded probes could not be visualised under AFM due to the strands being flexible. However, when each probe was hybridised with an RNA target, the strands’ structure altered to a stiff V-shape making it visible under AFM. Visualisation intensity also depended upon the position of each probe on the tile, for example, the centre compared to the edge of the tile. Between a concentration range of 10 nM and 200 pM, the LOD was calculated at 200 pM making this device not as sensitive as desired. Despite that, this work demonstrates how the precise and programmable arrangement of probes on a DNA origami scaffold increases detection by ensuring target molecules bind in well-defined orientations, producing detectable conformational changes and improving the effectiveness of AFM-based biosensing.

DNA has also been able to be detected through this biosensor. Xiong et al. combined DNA origami with AFM to determine repetitive DNA sequences [125]. A range of DNA origami shapes were created (triangle, cross, rectangle and nanotube) to act as nano-tags that consisted of a ‘tri-block’ to allow the repeated sequences to easily be determined by the DNA origami. This connection between the nanostructure tags and the repetition genes meant that they could be discovered due to their sequences and position. The nano-tags were tested on linear and circular DNA. By utilising AFM, precise locations of multiple repeated sequences were found at an extremely high resolution of 6.5 nm where the multiple repeats were discriminated easily between each other. This enhanced performance arises from the programmable and spatially controlled nature of DNA origami, which allows precise tagging and positioning along DNA strands, thereby improving resolution, specificity, and overall sensing accuracy. This system overall is ideal due to its low complexity, high precision, low time and cost effectiveness.

There are two main issues with utilising AFM in biosensing [126]. The first noticeable one is that it is costly. The AFM machine is known to cost thousands of pounds, and the probes extra hundreds of pounds. The probes themselves do not have unlimited use and, furthermore, can degrade over time which can lead to a large sum of money being put down on this device, especially if high-quality probes are going to be purchased. The next downfall of this biosensor is that the sharp probe can damage the samples when they come into contact. This can lead to indents, scratches and deformities, specifically if the target substrate is delicate. This interaction can lead to false results being produced and misleading images being visualised. A summary of AFM-based biosensors that utilise DNA nano origami is summarised in Table 5.

### 2.8. Wearable Biosensors

**Figure 7:**
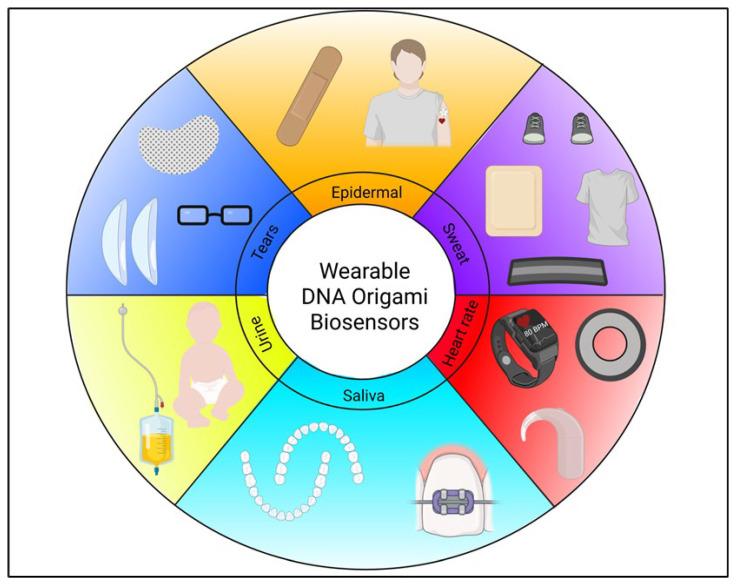
Wearable DNA origami biosensors for non-invasive health monitoring. Integrated into wearable devices, they detect biomarkers in saliva, urine, tears, sweat, and on the skin, while also monitoring physiological signals such as heart rate. Their versatility supports real-time, point-of-care diagnostics. Created in BioRender. Created with BioRender.com.

A rapidly growing type of analytical biosensing device that has started to gain attention is the wearable biosensor. These sensors are made to be worn on or near the body so that real-time, continuous data can be produced by monitoring physiological and biochemical analytes [127]. They have the same key features as in vivo biosensors; however, they are non-invasive or minimally invasive which makes them more attractive [128]. They are also created to look as natural and comfortable as possible, taking the forms of contact lenses, smart watches and even items of clothing [129] to monitor bodily fluids such as sweat, tears, saliva, and urine [130] (Figure 7). With further incorporation of AI, smartphones can be compatible with these biosensors to make them more personalised and user-friendly for each individual who will use these devices [131].

Even with the huge range of wearable biosensor platforms that have been researched [132,133,134,135,136], the direct integration of DNA origami (as opposed to other DNA-based materials) into wearable sensors remains limited in the literature. This is not to say that research is not actively being conducted in this area. Many scientists are already predicting that wearable biosensors with DNA origami will be the future of biomedicine [108,137] and in the coming years there will be a range of research accessible for everyone to read.

## 3. Tools Used to Design DNA Origami

### 3.1. Bottom-Up Approach

When it comes to designing DNA origami shapes and structures, the most common practice is the bottom-up approach, which typically uses computer-aided design (CAD) software to plan how staple strands route along a long ssDNA scaffold. Common scaffolded DNA origami CAD tools include caDNAno [138], scadnano [139], Tiamat [140], ENSnano [141], and GIDEON [142]. In addition, some tools focus on routing and optimisation, such as K-Router [143]. Other software supports post-design conversion and interoperability (e.g., exporting designs into simulation-ready formats), such as tacoxDNA [144]. There are also broader DNA nanostructure design frameworks and pipelines, including CATANA [145] and MOSES [146]. Finally, closely related nucleic acid design tools exist for other modalities, such as ROAD, which is used specifically for RNA origami design [147]. These software packages are widely used because they allow users to design DNA origami by selecting scaffold routing and staple placement, producing a full list of staple sequences required for experimental self-assembly [18].

To explain this approach in more detail, this software gives the opportunity to design specific 2D and 3D structures from scratch where scientists may not have prior experience in engineering their own customisable DNAs [21]. The length of the scaffold is determined in a lattice due to the fact that the choice of lattice has a huge influence on the final shape of the origami. For example [148], a honeycomb lattice allows each DNA helix to be arranged in a hexagonal pattern where they are allowed up to three further helices to be in a neighbouring distance at a 120° angle, whereas a square lattice allows each DNA helix to be arranged in a rectangular pattern where they are allowed up to four further helices to be in a neighbouring distance at a 90° angle. A honeycomb lattice is ideal to create more complex 3D designs such as nanotubes; however, a square lattice is favourable when creating flatter surfaces such as a sheet of paper. After the scaffold has been selected, complementary staple strands are placed in certain sections that create crossovers between the individual staples and the specific locations on the scaffold [149]. By creating these crossovers, it encourages the structure to fold specifically and remain in its intended shape. This software is a great tool at this point as it can generate ideal sequences for the staples so that they remain complementary to the scaffold. Once users are satisfied with their design, they are able to visualise what they have designed [150]. This can be done by showing a predicted image of the shape they have created, the sequences the software has generated and the length of each individual sequence. This makes it easier to determine what the design of the nanostructure will be, and simpler when ordering the staples as the user will have the exact list they need to create their shape.

Once all the sequences are known and have been purchased, DNA origami self-assembly can begin. This process works due to the complementary DNA sequences that were designed using the computer-aided software, and that can bind together by undergoing the Watson–Crick base pair rule in thermal annealing conditions [151]. This process focuses on heating and cooling a mixture that contains the long scaffold and the designed staple sequences in an appropriate buffer mixture which can stabilise the nanostructure by neutralising the negatively charged DNA [152]. This mixture is then heated to a high temperature to denature any pre-existing bonds between the dsDNA to ensure that all the strands are single-stranded in the mixture [153]. The mixture is finally cooled slowly (to room temperature or 4 °C) over several hours or days to ensure that the staple strands bind accurately to their complementary section on the scaffold to enable effective folding of the shape.

After self-assembly has occurred, the DNA origami mixture is purified to separate any excess material from the desired DNA origami shape, which can be done with a large range of techniques such as agarose gel electrophoresis, ethanol precipitation, glycerol gradient ultracentrifugation, PEG precipitation, spin filtration, size exclusion chromatography and SPRI beads [154].

Once purification has been conducted, the DNA origami is visualised under a microscope, transmission electron microscopy (TEM) or AFM, to confirm their shape and structure [155]. This is the final step in the bottom-up approach.

This approach allows researchers to engineer a wide range of precise nanoscale designs in a straightforward manner. The computer-aided software available is extremely accessible, and the entire process is kept simple by having access to ordinary experimental designs [156]. However, this approach is limited by the accuracy of the folding, as even though it is rated very well, achieving perfect folding 100% of the time is not possible and still remains a big challenge, especially with more complex structures [157].

### 3.2. Top-Down Approach

Another way DNA origami shapes and structures are designed is through the top-down method. This approach is not done very often but when it is it involves inputting the final desired shape into software that can generate the scaffold and staples independently to create the image in either a 2D pattern or a more complex 3D shape [158]. These platforms translate the chosen shape into DNA geometry by converting the design into the DNA helices, calculating the length of the ssDNA scaffold that is required and even pinpointing the most appropriate places for the crossovers between the helices and the scaffold to create complementary staple strands so that the shape can be folded accurately to the imported structure. Also considered are the physical characteristics of DNA so that all the angles and spacing produced is as accurate as possible.

Some of the available software that can be used for the top-down approach are: SARSE [159], FOLDNA [160], BSCOR [161], vHelix [162], Adenita [163], DAEDALUS [164], TALOS [165], PERDIX [166], METIS [167], ATHENA [168], oxView [169] and DNAforge [170].

Once the software generates the ideal complementary sequences, the scaffold and staples can be purchased online, assembled through annealing, purified and visualised in exactly the manner as the bottom-up approach [151,152,153,154,155].

This top-down approach, when it comes to designing DNA origami using the software, is advantageous because it supports almost any 2D and 3D arbitrary nanostructure; it automatically routes the scaffold and generates the appropriate staples, it is able to scale up to very large structures, it allows the integration of other molecules (gold nanoparticles, aptamers and proteins), it reduces the labour time and it is able to minimise human error [171]. However, this top-down approach lacks in credibility as it is not user-friendly due to the lack of tutorials available for the software programs, many of which require external programs (with some calling for multiple) to operate at their full potential, and they are still able to produce routes that are difficult to fold leading to structures that have low folding yields [172].

### 3.3. Simulation Approach

A final approach that is used to help design DNA origami is simulation tools. This concept is used in DNA nanotechnology because it can help predict how more complex shapes and structures behave, fold, and stay stable in real-world applications [172]. Due to the fact that DNA origami nanostructures contain thousands of complementary base pairs that are arranged in a specific way, it can be very hard and time-consuming to validate the accuracy of their shapes through human experiments. By using these computer-aided tools, this process can be sped up by providing the most optimal solutions before synthesis occurs [173]. Simulation tools can also assist researchers by reducing the amount of time spent on trial and error that is dedicated to creating accurately folded and stable structures [174].

Some of the most popular simulation software programs are: CanDo [175], SNUPI [176], MrDNA [177], oxDNA [178], oxRNA [179], and oxNA [180].

These simulation tools are beneficial due to the fact that they offer researchers the ability to predict key behaviours of DNA origami before conducting experiments, allowing the entire process to be sped up. They are extremely advantageous when it comes to developing larger, more complex 3D structures which initially would have been time-consuming and extremely costly to determine the characteristics of without these frameworks [171]. However, simulation tools are limited by their coarse-grained modelling which limits the accuracy of what can occur in real-life biological systems. This is because the software simplifies the complex molecular systems, specific interactions, solvent effects, and electrostatics [181]. Bias can also arise in parameterisation as the modelling systems used may not be as accurate as intended, due to the fact that this concept focuses more on a subjective decision regarding parameters rather than a factual objective set of criteria, further limiting the accuracy [182]. In addition to this, there is inaccuracy in the folding predictions as simulations prioritise the equilibrium states of the nanostructures rather than the assembly kinetics, which means the final stable conformation of the folded shape is not taken into consideration—only the rate of how fast the folding assembly is going to occur [183].

Table 6 summarises the three types of approaches mentioned in this section that help design DNA origami.

### 3.4. AI and Machine Learning in DNA Origami Design

The use of AI and machine learning to design DNA nanostructures has also been utilised recently due to their power to automate and optimise DNA design and reduce the probability of misfolding DNA nanostructures. Truong-Quoc et al. developed a graph neural network-based framework (DGNN) to predict the 3D equilibrium shape of DNA origami structures directly from design information [184]. The model represents each base pair as a node and encodes both local structural features and longer-range electrostatic interactions. To address the limited number of validated DNA origami datasets, the authors combined a data-driven loss with a physics-informed loss, allowing the model to learn from both labelled and unlabelled designs.

Singha et al. recently demonstrated a fingerprinting nanosensor array integrated with machine learning (ML) to distinguish between DNA origami shapes [185]. The nanosensor array was able to distinguish between triangle and nanotube shapes as well as differentiate them from an unfolded scaffold strand. Zubia-Aranburu et al. developed a dynamic light scattering (DLS)- and machine learning-based approach to predict DNA origami stability under physiologically relevant conditions [186]. Their results highlight strong shape-dependent effects on stability and suggest that data-driven models can support the rational design of more robust DNA origami systems for biomedical use. In terms of characterisation, AI has been demonstrated with a recent paper [187]. Researchers developed a transfer learning framework to classify DNA origami nanostructures in TEM images using convolutional neural networks. The study combined a larger simulation-derived image set for pretraining with a smaller experimental TEM dataset for fine-tuning, allowing more reliable characterisation despite limited experimental data. All these approaches could allow for the improved, rapid and robust design of DNA nanostructures for biosensing applications.

## 4. Conclusions

It is no surprise that biosensors continue to be adapted to become more dependable to this day. Their highly sensitive, user-friendly, fast-testing, versatile, real-time detection devices make them advantageous for point-of-care and detection testing. The integration of DNA origami makes them even more attractive because it allows the different types of biosensors to become more precise for different analytes due to their programmable nature. Their ability to spatially organise aptamers, nanoparticles, enzymes and fluorophores with nanometre accuracy provides an extraordinary amount of control when it comes to signal generation and molecular recognition. These models are also tunable due to the fact the specific biomolecular targets in play can easily be adjusted on the nanostructure to improve the overall sensitivity and specificity of the biosensor model. Having the choice to either design label or label-free biosensors allows a huge range of targets to be detected, such as metal ions, small and large organic molecules, bacteria, viruses and toxins. They are also capable of multiplex detection so that a range of analytes in the same category can provide a visual signal simultaneously. This opens up multiple fields to allow diagnosis to be recognised, such as the medical sector and the environment as a whole. The incorporation of wearable biosensors in this field, as well as the inclusion of AI, will expand DNA origami biosensors by making them mainstream not only in the field of nanotechnology research, but also to the general public.

However, despite these advances, DNA origami biosensors face several challenges that limit their practical application. The main bottleneck that is preventing DNA origami from replacing conventional biosensors is its vulnerability to nuclease degradation, as it directly affects the stability and reliability of the biosensors in physiological environments. Photobleaching can compromise signal detection and reproducibility remains difficult, especially with larger, more complex samples. In addition, practical issues such as high manufacturing complexity and associated costs further prevent large-scale adoption. To address these issues, future research should focus on developing technical strategies such as nuclease-resistant DNA designs, protective coatings, chemically modified nucleotides, and crosslinking to enhance structural stability against nucleases. As well as that, improving assembly protocols and creating high-fidelity software can enhance reproducibility. Looking ahead, AI-assisted signal analysis can help ensure reliable detection in complex samples while integration into wearable devices can facilitate practical application. Beyond signal analysis, AI and machine learning are expected to play an important role in the design and optimisation of DNA origami nanostructures, enabling smarter predictive modelling of structural behaviour and target interactions. In the future, the integration of CRISPR-based biosensing within DNA origami sensors could introduce the potential for collaborative sensing systems, where multiple detection elements can operate collectively to enhance sensitivity, specificity, multiplexing capabilities and precise spatial organisation to improve DNA origami performance. Overcoming these challenges will enable DNA origami biosensors to progress from experimental models to practical tools for early diagnostics, point-of-care testing, and real-time environmental monitoring, bringing them closer to replacing conventional biosensor types.

## Figures and Tables

**Table 1:** Chronological development of biosensors from 1962 to the present year. The table highlights significant milestones, including early enzyme-based sensors and immunosensors, the introduction of SPR, miniaturised biosensors, real-time devices and the recent advancement of wearable AI-incorporated biosensors.

Year	Invention/Development of Biosensor	Description
1962	First enzyme-based sensor	Detects glucose by utilising glucose oxidase to detect the changes in the consumption of oxygen [3].
1969	First urea-based sensor	Uses a potentiometric system that measures ammonium ions produced by the urease enzyme [4].
1975	First commercial benchtop biosensor	Directly measures the concentration of glucose from blood or plasma samples [5].
1975	First immunosensor	This used a microbe to detect ovalbumin [6].
1983	SPR	This was first introduced to biosensing with the application of detecting gas [7].
1984	First mediated amperometric biosensor was created	Used oxidative and reductive reactions to measure current that has been generated [8].
1990	The first commercial SPR biosensor	This was created by a company known as Pharmacia Biacore, which determined molecules’ characteristics through affinity and kinetics [9].
1992	The first commercial lab-on-a-chip blood biosensor called i-STAT	This was created by a company named Abbott Laboratories, which provided rapid results within minutes, whilst also being a handheld system [10].
1996	The first biosensor that incorporated aptamers—optical biosensor	This development was created by using fluorescent labelling [11].
2002	Electrodeposition paints	This was incorporated into biosensors to function as immobilisation matrices [12].
2003	Nanosensor plant implantation	This was embedded into a living plant to monitor a range of conditions [13].
2007	A real-time glucose electrochemical nanosensor	This was implanted into a subcutaneous adipose tissue for 5 days to determine its possibilities, and the results were accurate and consistent [14].
2008	The first DNA origami biosensor	This was the first reported DNA origami-incorporated biosensor that involved a 2D DNA rectangle nanostructure to detect RNA at specific locations [15].
2009	Graphene oxide in biosensors	The introduction of biosensors helped to create a better understanding in relation to fluorescence quenching [16].
2015	Wearable biosensors (smartwatches, mouth guards and contact lenses)	The most recent phenomenon is being developed by incorporating AI to monitor biochemical data from individuals’ bodily fluids to aid in detecting health-related biomarkers. Machine learning stored in these devices, in theory, is capable of real-time monitoring of biofluids to help maintain stable levels and also assist in diagnosing patients [17].

**Table 2:** A summary of fluorescence-based biosensors utilising DNA origami (n.d: not determined).

Transducer	Analyte	LOD	LOQ	Linear Range	Advantages	Disadvantages	Reference
Fluorescence	microRNA 30c, microRNA 223	0.4 µM	n.d	0.4–1.6 µM	Measurable FRET signal. Controllable. Reduces non-specific binding. Increases sensitivity. Increases reliability. Reusable.	Photobleaching. Colour bleed-through.	[33]
Fluorescence	Prostate-specific antigen (PSA)	30 pg/mL	n.d	200 pg/mL to 300 ng/mL	Measurable FRET signal. Increases specificity. Controllable. Increases sensitivity.	Photobleaching. Colour bleed-through.	[34]
Fluorescence	ATP	1 mM	N/A	0.10 mM to 1.00 mM	Measurable FRET signal. Increases sensitivity.	Limited sensitivity. Photobleaching. Colour bleed-through.	[36]
Fluorescence	Mercury ions	1.78 nM	2.0 nM	1 to 1000 nM	Measurable FRET signal. Works in real time.	Photobleaching. Colour bleed-through.	[40]
Fluorescence	Mercury, silver and lead ions	10 to 20 nM	n.d	10 to 2000 nM	Simultaneous detection. In-depth analysis. Increases reproducibility. Increases reliability. Cheaper. Controllable.	Cross-reactivity. Limited sensitivity. Limited specificity. Increases S/N ratio. False positives.	[118]
Surface-Enhanced Fluorescence	Carbapenem-resistant Klebsiella pneumoniae	5 aM	n.d	1 aM to 5 nM	Increases sensitivity. Controllable.	Photobleaching and colour bleed-through.	[52]

**Table 3:** A summary of surface-enhanced Raman resonance (SERS)- and surface plasmon resonance-based biosensors utilising DNA origami. (n.d: not determined).

Transducer	Analyte	LOD	LOQ	Linear Range	Advantages	Disadvantages	Reference
SERS	TAMRA–DNA	S/N of 5.8	n.d	n.d	Increased sensitivity. Controllable. Increased reliability. Increased reproducibility.	Difficult to maintain reproducibility and reliability.	[59]
SERS	Epidermal growth factor receptor (EGFR)	0.2 nM	n.d	20 nM to 20 pM	Increased sensitivity. Controllable. Increased reliability. Increased reproducibility.	Difficult to maintain reproducibility and reliability.	[66]
SERS	Diethylstilbestrol	Ranged from 0.26 to 0.62 ng/mL	Ranged from 0.8 to 1.9 ng/mL	10^−10^ to 10^−5^ M	Increased sensitivity. Controllable. Increased reliability. Increased reproducibility. Recoverable.	Difficult to maintain reproducibility and reliability.	[67]
SERS	DU145 cells	20 pM	n.d	n.d	Increased sensitivity. Controllable. Increased reliability. Increased reproducibility.	Difficult to maintain reproducibility and reliability.	[69]
SERS	Thrombin protein	Single-molecule detection	n.d	n.d	Increased sensitivity. Controllable. Increased reliability. Increased reproducibility.	Difficult to maintain reproducibility and reliability.	[71]
SERS	HRP	Single-molecule detection	n.d	n.d	Increased sensitivity. Controllable. Increased reliability. Increased reproducibility.	Difficult to maintain reproducibility and reliability.	[72]
SPR	Haemoglobin, glycated haemoglobin	n.d	n.d	0.025–0.1 mg/mL	Controllable. Increased selectivity.	Non-specific binding. Increased S/N ratio. False positives. Not reliable.	[81]

**Table 4:** A summary of electrochemical and nanopore sensors utilising DNA origami.

Transducer	Analyte	LOD	LOQ	Linear Range	Advantages	Disadvantages	Reference
Electrochemical	Streptavidin	Streptavidin: <1 pM	n.d	5 pM to 1 nM	Simple to operate. Cheap. Easily portable. Short detection time. Regenerative.	Decreases stability. Increases interference. Poor selectivity.	[87]
Nanopore	holo human serum transferrin	Single-molecule detection	n.d	n.d	Controllable. Increases sensitivity.	Decreases stability. Increases interference. Poor selectivity. Difficult to control speed of translocation.	[96]
Nanopore	ribonuclease A, carbonic anhydrase, ovalbumin, avidin, dCas9, ClpP	Single-molecule detection	n.d	n.d	Controllable. Increases sensitivity. Increases S/N ratio. Increases trapping.	Decreases stability. Increases interference. Poor selectivity. Difficult to control speed of translocation.	[97]
Nanopore	miRNA-141-3p	n.d	n.d	0.2–200 nM	Controllable. Increases sensitivity. Increases reliability.	Decreases stability. Increases interference. Poor selectivity. Difficult to control speed of translocation.	[99]

**Table 5:** A summary of selected atomic force microscopy (AFM)-based biosensors utilising DNA origami.

Transducer	Analyte	LOD	LOQ	Linear Range	Advantages	Disadvantages	Reference
AFM	Rag-1, c-myc, and β-actin mRNA	200 pM	n.d	n.d	Label-free, highly specific, nanoscale-resolved RNA detection with programmable probe placement and multiplexing capability.	Dependence on AFM imaging, low throughput, and the lack of a simple real-time quantitative readout compared with conventional amplification-based assays.	[15]
AFM	miRNA-155, miRNA-182, miRNA-197	n.d	n.d	n.d	Simultaneous detection. In-depth analysis. Increases reproducibility. Increases reliability. Cheaper. Reduces false positives. Controllable. Highly programmable.	Cross-reactivity. Limited sensitivity. Limited specificity. Increased S/N ratio. False positives.	[118]

**Table 6:** Summary of approaches used to design DNA origami. This table provides an overview of the three main approaches used in DNA origami design. It describes the main reasons as to why this tool is used, as well as the advantages and disadvantages of using each approach to create nanostructures.

Approach	Description	Advantages	Disadvantages
Bottom-up	Users create their 2D or 3D DNA origami structures manually by choosing where staples and crossovers are specifically placed on a long ssDNA scaffold. Once generated, the complementary sequences, scaffold and staples are purchased online, assembled through annealing, purified and visualised through experiments.	Accessible to create a wide range of precise 2D and 3D nanoscale designs, user-friendly and involves standard laboratory techniques.	Accuracy of the folding is limited especially with more complex structures.
Top-down	The desired shape is imputed into software to generate the scaffold, staples and crossover points to create 2D or 3D nanostructures. Once the software generates the ideal complementary sequences, the scaffold and staples are purchased online, assembled through annealing, purified and visualised through experiments.	Supports almost any 2D and 3D arbitrary nanostructure, automatically routes the scaffold and generates the appropriate staples. Compatible with scaling up to very large structures, it easily integrates other molecules, reduces labour time and minimises human error.	Not user-friendly, often requires external (and sometimes multiple) programs to operate effectively, and some routes produced can be difficult to fold leading to low folding yields.
Simulation	Help predicts how more complex shapes and structures behave, fold and stay stable in real-world applications by providing the most optimal solutions before synthesis occurs.	Most beneficial when developing larger, more complex 3D structures, reduces trial and error, reduces costs, and saves time.	Uses coarse-grained modelling which limits the accuracy of real-life biological systems. Parameter bias can arise as well as inaccuracy in kinetic folding predictions.

## Data Availability

No new data were created or analysed in this study.
